# Striving towards the ideal cardiac functional assessment strategy: the contribution of tissue Doppler, strain and strain rate imaging

**Published:** 2007

**Authors:** JC Moolman-Smook, PA Brink

**Affiliations:** Department of Biomedical Sciences, Faculty of Health Sciences, University of Stellenbosch; Department of Medicine, Faculty of Health Sciences, University of Stellenbosch

## Abstract

In cardiac research, a major goal of prevention of catastrophic events by risk-factor management and earlier detection has, in recent years, led to a proliferation of imaging modalities, moving us from old-fashioned chest X-ray through increasingly sophisticated approaches such as magnetic resonance imaging (MRI) and multi-slice fast computer-aided tomography (CT) scanning. Today, we have the option of using a vast array of invasive and non-invasive approaches, with diverse technical underpinnings, to assess various, and often overlapping aspects of cardiac function.

Tissue Doppler imaging (TDI) and the related applications of strain and strain rate imaging are new technologies that are now being evaluated in the realm of practical patient care, and the underlying principles remind us that cardiac contractility is a reflection of the integration of muscle fibre architecture, mechanics and metabolism. TDI is the first technology that allowed imaging of motion within the myocardial wall rather than that of the blood pool, and permits analysis of velocities and accelerations from ultrasonic scatterers in muscle.^1^ Since its inception, it has been used to evaluate both new cardiac functional parameters as well as conventional function; for some of these, TDI has proven the superior imaging modality, while for others it offers only incremental information over conventional approaches.

## From the single fibre to the whole heart and segmental function

The efficiency of the cardiac pump depends on the effective contraction and relaxation (contractility) of the muscle walls and the topology and geometry of the cardiac chambers. It is not a homogeneous muscle mass, but its molecular constitution varies; this includes both normal variation and variation arising from disease states, which presumably affects contractility. These differences in constitution and architecture are seen between the endocardium, mesocardium and epicardium, between the left and the right ventricle as well as within different localities in these chambers, between young and older individuals, between normotensive and hypertensive states, and between diseases such as ischaemic heart disease and cardiomyopathies.

Our perceptions of cardiac contractility have to a great extent been shaped by isolated heart muscle fibre experiments (for overview see references 2 and 3). By extrapolating these findings to the whole ventricle, a number of concepts have evolved.

● Velocity and power of contraction in single fibres are directly related to fibre length at the beginning of contraction (also known as the Frank-Starling law). For the whole heart, this is extrapolated to consider the geometry of the heart, so that velocity and power of contraction are generally correlated with ventricular volume or diameter at the end of diastole.
● During contraction in single-fibre experiments, the increasing tension is taken up by in-line elastic components and possibly by elastic components operating in parallel. Force is generated by contractile elements called sarcomeres, and involves thickening in some places and thinning in others. There is an isometric phase during which there is no fibre shortening, and is equivalent to the isovolumic phase, while the shortening phase correlates with the ejection phase of systole. Similar equivalents exist for early and late diastole.
● The tension and velocities generated in either single fibres or the whole heart can be manipulated by physical parameters such as starting length or load, and pharmacological and metabolic means.

Terminology from such single-fibre experiments has led, at the level of the whole heart, to concepts of the rate of pressure development (dP/dt), wall tension, preload, afterload, end-systolic pressure–volume relationship (elastance), inotropic state, etc. Measurement of fractional shortening and ejection fraction became a ‘surrogate’ for pump function in the absence of direct information regarding pressure development from all the noninvasive diagnostic methods.

Furthermore, anatomical and histological studies have taught us that the muscle of the heart is layered in a spiral fashion. While subendocardial fibres, which have a mostly lengthwise orientation, are mainly responsible for longitudinal shortening of the heart, subepicardial fibres, with a more circumferential orientation are mainly responsible for radial (short-axis) contraction. Ejection is therefore not just the result of inward pressure mounted from concentric forces when these muscle layers start to tense, but, very importantly, longitudinal shortening of the muscle fibres combined with these concentric forces results in a twisting effect, like a wet cloth being wrung out. Of course, the reverse happens in diastole. During cardiac contraction, the apex remains fixed, while the base of the heart ‘screws down’ onto the apex, with, as we have learnt from TDI, a velocity gradient running from base to apex.

Reasoning from an anthropological principle, this whole sequence is purposeful, and in aspiring to assess myocardial function, we should strive to take account of these known facts. The ideal approach to assessing cardiac function would offer non-invasive measurement of the velocity and rate of acceleration of myocardial regions, as well as the moment-to-moment changes in chamber pressures. Currently no imaging modality can offer these features, but TDI, strain and strain rate imaging do at least address the issue of velocity and rate of acceleration of myocardial regions, and can give an indirect indication of tension and force development derived from measurements of conformational changes within muscle.

## Principles of tissue Doppler imaging

The normal myocardial fibre architecture described earlier gives rise to differences in velocities of contraction in distinct myocardial regions, which can be detected by TDI. In contrast to conventional Doppler echocardiography, which produces blood flow data dependent on velocities of elements within the blood, TDI quantitatively measures velocities of elements (‘scatterers’) within the myocardium itself. Relative to blood flow, muscle movement (0.06–0.24 ms) is 10 times slower and returns high-intensity echoes (40 decibels higher than blood).^4^ By means of a so-called ‘wall filter’, and facilitated by the highest capacity for frame rates of any current imaging modality, TDI can discriminate between muscle movement and blood flow, and can detect short-lived motion usually undetectable by the human eye. This allows TDI to record transient events in the cardiac cycle, including the isovolumic stages and post-systolic shortening.

As with blood-flow Doppler, TDI accuracy depends on alignment of the probe with the elements generating the velocity, ie, with the direction of wall motion. However, in contrast to conventional grey-scale echocardiography, velocity data does not depend on the amplitude of the reflected signal, but rather on shifts in frequency. Therefore, because it is not absolutely dependent on a clearly defined border, and because data on myocardial movement can be obtained from an extremely small segment (1 × 1 mm on GE Healthcare’s Vivid-7), even suboptimal echocardiographic images can be assessed with TDI.^4^

Two types of TDI are currently available, pulse-wave and colour TDI. The first allows immediate analysis of muscle movement, which displays peak velocities at single locations (with particular functional implications), but does not allow comparison across myocardial regions. On the other hand, colour TDI can be used to detect velocity differences between the endo- and epicardium, from which the myocardial velocity gradient can be derived.

An important limitation in interpreting such TDI results is that the velocities measured are determined not only by contraction and relaxation of the fibres themselves, but also by movement of the heart within the chest and the twisting motion of the heart when it contracts (so-called translational effects). Newer applications of colour TDI make use of deformation measurements (strain and strain rate) where particular myocardial velocities are correlated with other local velocities, making them relatively independent of translational effects and facilitating functional evaluations across myocardial regions. With colour TDI, strain and strain rate, a mass of imaging data are collected reasonably fast in a formulated manner. These images are then analysed off-line, which, although time consuming, has advantages and disadvantages.^5^,^5^

## Strain and strain rate imaging

In strain and strain rate imaging, myocardial strain is defined as the deformation of a section of myocardium in comparison with its original size and form. In a sense, shortening fraction and ejection fraction represent a gross form of strain; similarly, myocardial strain is dimensionless and usually shown as a percentage. Strain rate refers to the deformation of the region of muscle over time.

Strain rate can currently be measured in only one dimension, which implies that the sonar beam must be parallel or perpendicular to the direction of deformation. If it were possible, three-dimensional strain imaging would be able to measure deformation in nine directions, which would then include the effect of shearing forces, namely, forces operating in directions not aligned with the main force.

Currently, this is not possible with any echocardiography techniques, but is feasible with MRI. However, MRI is time consuming, acquisition of data takes longer and facilities are less readily available.^6^ Moreover, the strain measured in colour TDI reflects ‘natural strain’,^6^ which is based on instantaneous dimensions. It is not dependent on knowledge of the initial dimension, and is different from the strain measured by MRI, which is in essence Lagrangian strain. Although these two types of strain are similar at the level of 5 to 10% deformation, they are quite different at high percentages. In TDI-based strain imaging, strain rate can be derived from tissue velocities, while strain is calculated as the spatial integral of strain rate.

Strain and strain rate imaging are new developments and the technology is currently being further refined. TDI-based strain and strain rate are extremely sensitive to the direction of the transducer. This drawback has been overcome by another new and related technique, namely, speckle-tracking imaging (STI), which is used to measure tissue velocity, strain and strain rate. In STI, speckles that are constant features within the myocardial image are tracked frame by frame, and yield information about muscle deformation in two orthogonal directions, both longitudinal and radial dimensions.^7^

If strain and strain rate imaging could be extended to three-dimensional analysis, it would make this modality as informative as MRI, but with the added advantage of easier data collection. The applications of strain and strain rate imaging in day-to-day patient care are still being explored. While strain correlates with changes in stroke volume, and therefore with global contractility, strain rate seems to carry the same information as rate of pressure development in the ventricles (dP/dt).^8^ Currently, its most significant application is in the assessment of patients for cardiac resynchronisation therapy. In contrast, the applications of particularly pulse-wave TDI in routine clinical practice are well established.

## Current clinical applications of TDI, strain and strain rate imaging

Performance of any new technology should be compared with existing gold standards before its practical utility and its ability to improve on or replace existing technologies can be interpreted, and, accordingly, the application of TDI to measure numerous aspects of systolic and diastolic function has been assessed over the past decade or so.

Longitudinal shortening of the subendocardial fibres of entire cardiac walls, which is the primary event in cardiac contraction, is reflected in the displacement of mitral and tricuspid valve annuli, and this displacement is therefore considered a good surrogate for global left or right ventricular function. It is typically assessed by averaging mitral or tricuspid annular displacement velocity measured by pulse-wave TDI at two or more of the corners of valve annuli (lateral, septal, anterior and inferior) during systole and diastole. This value derived from mitral annular displacement in systole (Sa) has been shown to correlate well with left ventricular ejection fraction (LVEF) determined by two-dimensional echocardiography (Sa < 7 cm/s indicates LVEF < 45%).^9^

Diastolic function has conventionally been assessed by blood flow through the mitral valve in conventional Doppler echocardiography. However, these Doppler flow variables are highly load and volume dependent, making them inaccurate measures of diastolic function for some patients, such as those with hypertrophic cardiomyopathy (HCM) or mitral valve disease. By contrast, early diastolic mitral annular displacement velocity (Ea) may be less dependent on preload.^10^,^11^ The ratio of conventional Doppler early diastolic mitral valve flow (E) to Ea (E/Ea) of ≥ 10 has been shown to correlate well with mean left ventricular end-diastolic pressure ≥ 15 mmHg, irrespective of the LVEF or the presence of mitral regurgitation.^12^,^13^

Another TDI parameter, namely tissue Doppler (TD) MPI, which is related to the myocardial performance index (MPI) or Tei^14^ index, a Doppler-derived blood-flow measure of global ventricular function, has also been found to inversely correlate well with end-systolic elastance in an animal study.^15^ However, TD MPI is sensitive to loading conditions, and further studies in humans would be required to determine whether the loading conditions typical in human patients would preclude the utility of this approach.

The interrelationship of velocities generated from the distinct myocardial regions is often perturbed due to cardiac disease states well before manifesting via the conventional clinical or Doppler echocardiography parameters. Therefore, TDI measurement of systolic and diastolic function of both ventricles and atria can be used for earlier diagnosis, prognosis and risk stratification in increasing numbers of cardiac disorders, in some respects adding, although incrementally, to conventional parameters, and in other respects, facilitating non-invasively, measurements which otherwise would have required invasive approaches. Some of these are discussed briefly below.

## Non-invasive assessments

In addition to facilitating non-invasive estimation of filling pressures during assessment of left ventricular diastolic dysfunction, TDI may provide a more accessible and less invasive approach for measurement of right ventricular as well as atrial function.

*Right ventricular function:* To date, estimation of right ventricular function, which has prognostic and clinical value for heart failure and post-MI patients, has not been accurately assessable by conventional echocardiographic approaches. This is mainly due to imaging difficulties ensuing from right ventricular anatomy, so that cardiac MRI or CT imaging, which are generally less widely available, and introduce additional limitations, have had to be relied on. TDI may offer a fast, noninvasive approach for assessing right ventricular function, as peak systolic tricuspid annular velocity (Sa) < 11.5 cm/s has been shown to correlate well with right ventricular EF < 45%, and has been used in prognostication of heart failure patients.^16^,^17^

*Atrial function:* Atrial electrical function can only be precisely measured by invasive electrophysiological means, while atrial mechanical function is usually determined from atrial volume-derived indices, which are load and observer dependent. However, lately, a number of studies have demonstrated that TDI is a non-invasive, reliable and easy approach to estimate total atrial electrical activation time^18^ as well as mechanical function, independent of left ventricular function.^19^,^20^ Specifically, peak atrial systolic mitral annular velocity correlates well with left atrial systolic fractional area change and fractional volume change during atrial systole,^21^ while peak regional atrial contraction velocity [V(A)] may be a better reflection of atrial mechanical function than transmitral atrial velocity assessed by conventional Doppler echocardiography.^22^

## Early diagnosis

*Preclinical genetic disease:* Early identification of individuals at risk of genetically mediated cardiac disease is often desirable to facilitate implementation of preventive or remedial measures, however, variability in the age of onset and subtlety of preclinical features, as measured conventionally, may make clinical identification difficult. In addition, genetic identification can be complicated by heterogeneity of the causal genes and mutations. Because TDI can identify subtle signs of systolic and diastolic dysfunction, which cannot be detected by conventional twodimensional or blood-flow Doppler echocardiography, it can be used to identify cardiac involvement in sufferers of inherited disorders with a myocardial component, such as Friedreich’s ataxia, various muscular dystrophies and myotonic dystrophy, at an early stage of the disease.^23^

For example, the variability in the extent of left ventricular wall thickening and the age of onset of hypertrophy makes clinical identification of individuals at risk of developing HCM problematical. In HCM mutation carriers, both systolic and early diastolic velocities are markedly lower than in non-carriers (Ea < 13.5 cm/s), even before the development of hypertrophy. These reductions in velocities can be used to predict mutation carriers in HCM families, allowing cost-effective follow-up and intervention (Moolman-Smook, Brink, unpublished data).^24^-^27^

Another example is Fabry’s disease, which often presents with exclusively cardiac manifestations in female carriers and in male carriers of particular genetic mutations.^28^,^29^ Preclinical detection of cardiac involvement is highly desirable, as early enzyme replacement therapy can prevent the development of arrhythmic and thromboembolytic complications; however, conventional non-invasive approaches such as ECG, two-dimensional echocardiography and cardiac magnetic resonance imaging (cMRI) cannot do so.^30^

On the other hand, when compared to non-carriers, Fabry’s mutation carriers demonstrate marked reduction in TDI systolic and early diastolic mitral annular velocity, measured at lateral and septal corners of the mitral annulus, irrespective of the extent of left ventricular hypertrophy or other systemic manifestations. 31 In female Fabry’s mutation carriers, such findings may indicate the need for invasive assessment of cardiac involvement; on the other hand, the efficacy of enzyme replacement therapy can be monitored non-invasively by TDI.^32^

*Early graft rejection:* Cardiac transplant rejection is difficult to detect non-invasively at an early stage because of the non-specificity of conventional echocardiographic findings. However, in children with orthotopic heart transplants, TDI demonstrated a dramatic decrease in tricuspid (but not mitral) systolic and early diastolic velocities at three to six months before terminal graft failure. This has lead to the suggestion that bi- or tri-annual TDI examinations rather than annual invasive catheterisation and angiography may be a more efficient approach to survey for graft impairment.^33^,^34^

## Differential diagnosis

Although most conditions that affect myocardial function manifest with reduced myocardial velocities, a number of studies have demonstrated that various TDI indices, sometimes in conjunction with conventional clinical criteria, can provide discriminatory information for making differential diagnoses.

*Constrictive physiology:* For instance, in both restrictive cardiomyopathy and constrictive pericarditis, the heart manifests with abnormal left ventricular filling patterns; however, differentiating the two conditions is crucial for appropriate treatment. Respiratory variation in mitral and pulmonary flow on conventional Doppler echocardiography can differentiate constrictive pericarditis patients from those with restrictive cardiomyopathy in some but not all cases. However, when also using a peak TDI Ea of ≥ 8.0 cm/s, accuracy of diagnosis can be increased.^35^

*Hypertrophic states:* Similarly, differentiation of physiological hypertrophy, such as that seen in athlete’s heart, from pathological hypertrophy seen in disease states such as hypertension or hypertrophic cardiomyopathy, has implications for patient management and therapy. However, distinguishing these states by morphological information derived from conventional echocardiography is not clear-cut. TDI reveals that the long-axis systolic and early diastolic velocities are decreased in cases of pathological hypertrophy, but not in physiological hypertrophy.

The best differentiation of pathological hypertrophy (due to HCM, hypertensive heart disease) from physiological hypertrophy (athlete’s heart) was provided by a mean systolic annular velocity < 9 cm/s.^36^ Furthermore, patients with non-obstructive left ventricular hypertrophy due to HCM demonstrate greater heterogeneity of peak annular velocities, as determined from the median, lateral, inferior and anterior mitral annuli, which can discriminate this disease state from left ventricular hypertrophy due to hypertension.^36^,^37^ Similar discrimination between left ventricular thickening due to cardiac amyloidosis versus other causes of left ventricular hypertrophy can be made by a characteristically jagged myocardial velocity profile derived from TDI data.^38^

## Prognostication

Because TDI mitral annular velocities predominantly measure the long-axis contraction of the ventricle, they reflect the functionality of the subendocardial myofibres, which are particularly prone to disturbance by disease conditions. The extent of these disturbances, as reflected by altered velocities, is often predictive of the extent of the underlying condition, and therefore TDI myocardial velocities are useful predictors of outcome, adding incremental value to conventional prognostic indicators in a variety of cardiac conditions.^39^ A number of studies have shown that patients with hypertension, ischaemic heart disease, valvular heart disease, heart failure, diabetes and obstructive sleep apnea who present with an increased E/Ea ratio (> 20) have a significantly increased risk of adverse cardiac events.^40^-^42^

*Predicting responders to cardiac resynchronisation therapy:* Biventricular pacing is considered a promising technique for the treatment of congestive heart failure and dilated cardiomyopathy. Patients are selected for this treatment predominantly on the basis of a wide QRS. However, the therapy succeeds because it improves left ventricular mechanical synchrony, and therefore selection of patients on the basis of mechanical function of the myocardium may be more rational. Prepacing colour TDI assessment of 12 left ventricular segments is used to assign a dyssynchrony index for systolic function, which has proven very useful for predicting responders and non-responders to cardiac resynchronisation therapy (CRT).^43^ The TDI-derived dyssynchrony index has proven more useful than displacement or strain mapping^44^ although being comparable to velocity-encoded MRI,^45^ and facilitates cost-effective CRT by reducing the percentage of non-responders. TDI is also useful to define the degree and location of mechanical improvement subsequent to CRT, and can be used to guide pacemaker optimisation when CRT has proven less than optimal.^46^

*Prediction of preserved contractile function in MI patients:* Defining the degree of preserved myocardial function is clinically important after acute myocardial infarction. A TDI-derived peak systolic velocity at the mitral annulus of > 7.5 cm/s can be used to predict preserved global left ventricular systolic function with 79% sensitivity and 88% specificity in individuals who have suffered acute myocardial infarction.^47^

Furthermore, although the extent of the infarct can be defined by delayed-enhancement cMRI, such facilities are not always accessible. The absence of a TDI-measured positive pre-ejection velocity wave, which originates between ventricular activation and aortic valve opening^48^ correlates well with myocardial infarction-induced transmural necrosis, and therefore with absence of viability of myocardium.^49^ Similarly, TDI-based strain rate imaging can be used to differentiate between transmural [peak systolic strain rate (SRs) > −0.59 s^-1^] and nontransmural infarcts.^50^

In addition, quantitative pulsed-wave TDI assessment during a low-dose dobutamine challenge can differentiate between stunned, hibernating and irreversibly dysfunctional regions by the degree of reduction in regional myocardial wall velocities.^51^ However, dobutamine strain rate imaging may be more accurate for identifying hibernating myocardium.^52^

## Conclusion

TDI and the related applications of strain and strain rate imaging allow excellent quantitative assessment, specifically of focal myocardial function and are therefore second to none for estimation of diastolic function, optimal identification of individuals for cardiac resynchronisation therapy and early identification of sufferers of certain inherited cardiac disorders in the absence of genetic testing. TDI-related approaches have also turned out to be on a par with existing techniques for the non-invasive, indirect (by proxy) estimation of parameters pertaining to tension and force development, and only time will tell whether TDI will replace existing approaches for measuring these parameters. Certainly, for myocardial disease, colour TDI, strain and strain rate imaging are invaluable for opening up new possibilities to understanding disease pathology, and further technological development of these modalities, including STI, should be watched with interest.
